# The prevalence of premature thelarche in girls and gynecomastia in boys and the associated factors in children in Southern China

**DOI:** 10.1186/s12887-019-1426-6

**Published:** 2019-04-11

**Authors:** Jianwei Zhang, Jinliang Xu, Lifang Liu, Xiaohua Xu, Xinxian Shu, Zhifeng Yang, Lanqiu LV, Xiding Cai, Xianjiang Jin, Zaiyan Gu, Junfen Fu

**Affiliations:** 1 Department of Endocrinology, Children’s Hospital of Zhejiang University, School of Medicine, Zhejiang University, Hangzhou, China; 2 Shaoxing women and children hospital, Shaoxing, Zhejiang China; 3 Quality control center of children’s growth and development of Zhejiang province, Hangzhou, Zhejiang China

**Keywords:** Prevalence, Premature thelarche, Gynecomastia, Risk factors, China

## Abstract

**Background:**

To investigate the prevalence and risk factors of premature thelarche (PT) in girls and gynecomastia (GM) in boys in Southern China.

**Methods:**

We conducted a cross-sectional study of preschool children across 9 cities in Zhejiang province. A total of 6273 children in the age-group of 2–7 years were recruited from January 2014 to March 2015. Relevant information was collected from mothers through face-to-face interviews. Logistic regression models were used to examine the correlates of PT and GM. Odds ratios (ORs) with 95% confidence intervals (CIs) are reported.

**Results:**

The prevalence of PT among girls was 4.8% and that of GM among boys was 0.8%. One hundred girls were diagnosed with PT before the age of 2 years; 69 (69.0%) of these girls experienced spontaneous resolution of PT. Twenty-four boys were diagnosed with GM before the age of 2 years; 10 (41.7%) of these experienced spontaneous resolution of GM. Children borne of mothers with early onset of menarche and those belonging to high-income families were at a higher risk of premature breast development. Greater consumption of eggs was associated with premature breast development in early childhood.

**Conclusions:**

Socioeconomic status of family, early onset of menarche in mother, and consumption of eggs were strongly associated with premature breast development in early childhood.

## Background

Premature thelarche (PT) refers to isolated early breast development in girls younger than 8 years of age while gynecomastia (GM) refers to the presence of breast tissues in boys [1]. Surveys conducted across the world have shown an increasing trend in the prevalence of PT and GM among young children. A survey of 802 girls in Istanbul (year 2011) revealed an early breast growth rate of 8.9% among girls aged 8 years [2]. Similarly, the prevalence of PT among Italian and Danish girls under the age of 8 years was 11 and 3%, respectively [3, 4]. In a US national multicenter survey (year 1997) of 17,077 girls aged 3–4 years, 3% of African-American girls and 1% of white girls were found to have PT. [5] In a recent US regional study, the PT rate among 318 girls of age 1–4 years was found to be 4.7% [6]. In a multicenter study of 1510 Chinese infants and toddlers in the age-group of 0–4 years, the prevalence of PT and GM was found to be 2.2 and 1.0%, respectively [7].

Epidemiological evidence suggests that diet, environmental toxicity, and socioeconomic status of the family may be potentially associated with PT and GM in young children. Some studies have found an association of early breast development with consumption of phytoestrogen-containing food [8, 9] and endocrine disrupting chemicals [10, 11]. However, there is a lack of definitive evidence of this association. Some have claimed that the increased prevalence of obesity among children is responsible for early onset of puberty [2, 12, 13]. Other studies have investigated the influence of gestational and birth-related factors on pubertal onset; according to a study, prematurity may also be a risk factor for PT. [14]

China is a rapidly developing country with tremendous changes in socioeconomic and environmental milieu, especially in the southern areas. While a recent study found an increase in the incidence of PT and GM among Chinese children, robust studies with relatively large sample size are yet to be conducted. Therefore, in this study, we investigated the prevalence and correlates of PT and GM among Chinese children in the age-group of 2–7 years.

## Methods

### Participants

The data presented here were obtained from Zhejiang Investigation of Breast Development and Dietary Factors in Infants and Young children. This observational study included a retrospective survey and physical examination. This study was conducted in preschool children in 9 districts of Zhejiang province in Southeast China (Hangzhou, Ningbo, Shaoxing, Taizhou, Wenzhou, Jiaxing, Huzhou, Jinhua, and Lishui). In each district, children aged 2–7 years were randomly selected from a city and countryside preschool using a random number table from January 2014 to March 2015. A multistage, stratified cluster sampling technique was used to select the study sample. This study was approved by the Institutional Review Board of the Children’s Hospital of Zhejiang University and informed consent was obtained from the biological mothers of all children.

#### Questionnaire survey and physical examination

An interviewer solicited all relevant information from mothers via a face-to-face interview conducted using a specially designed questionnaire. Data pertaining to the following variables were collected in the interview: birth status (gestational weeks and birth weight); infant feeding status (feeding history during the first 6 months and 7–12 months after birth), history of diagnosis of early breast development by endocrinologist (whether the children were diagnosed with early breast development by an endocrinologist; whether it regressed naturally; duration for which the condition lasted); age of mother at menarche; food exposure; and family income. All children underwent physical examination conducted by trained pediatric endocrinologists; data pertaining to the extent of breast development, height, weight, waist circumference, and hip circumference were also collected. In this study, breast development was staged according to the Tanner criteria. Overweight children underwent breast ultrasound examination to assess the presence of early breast development. Children with early breast development were followed every 3 months until natural regression of the breasts or until age 8 if breast tissue persisted.

#### Statistical analysis

Data pertaining to continuous variables are presented as mean ± standard deviation; categorical variables are presented as frequency (percentage). Data pertaining to height, weight, waist circumference, and hip circumference were converted to the corresponding z values for age and sex. Logistic regression analyses were used to assess the risk factors of PT and GM and the corresponding odds ratios (ORs) with 95% confidence intervals (CIs) were calculated. The significance level was set at *P* < 0.05. All analyses were performed using the SPSS 20.0 statistical package.

## Results

### Prevalence and resolution of PT and GM

**Fig. 1:**
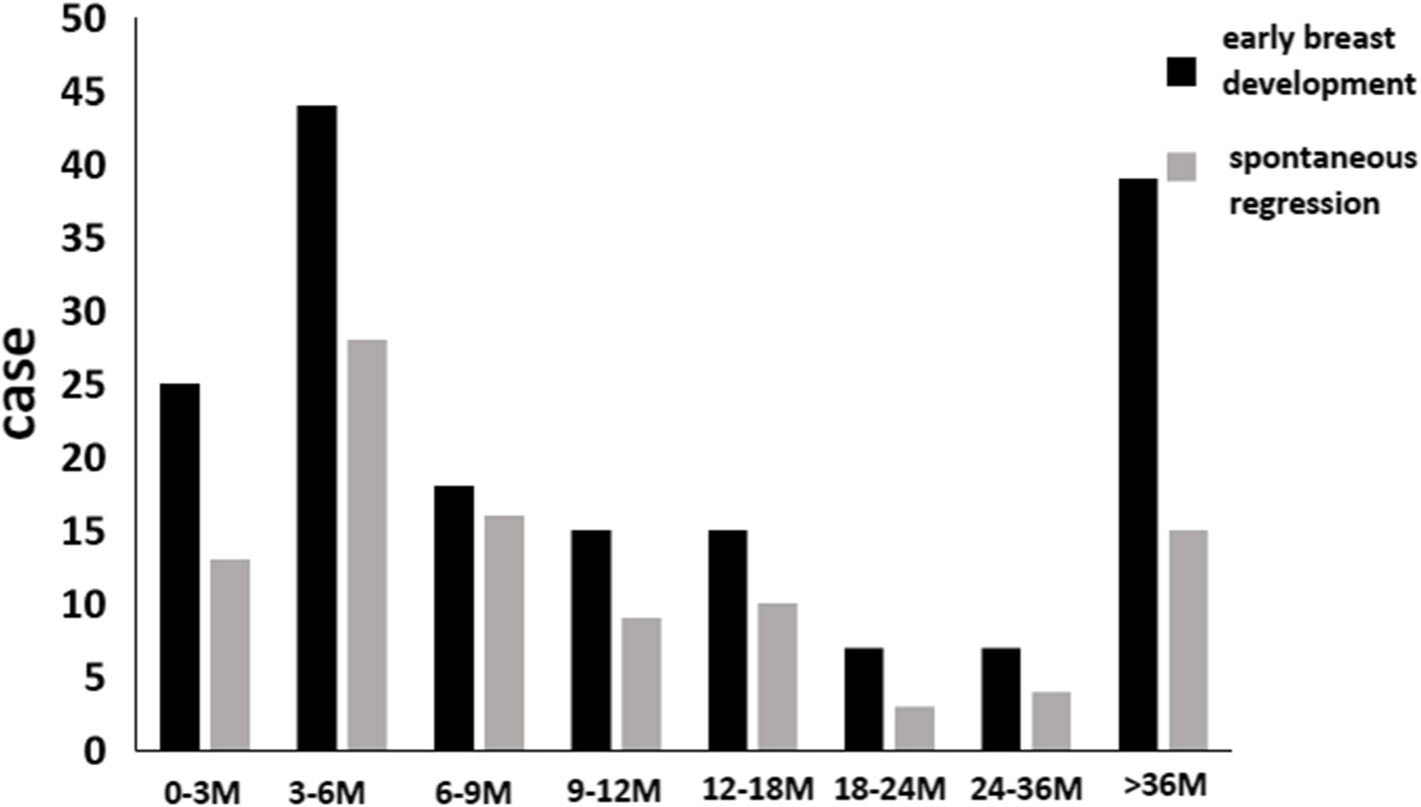
Age distribution of subjects who experienced spontaneous regression of premature thelarche and gynecomastia

A total of 6273 children (3295 boys and 2978 girls) in the age-group of 2–7 years were recruited in this study. The mean age of boys and girls was 4.01 ± 0.92 and 4.04 ± 0.97 years, respectively. Of these, 170 (143 girls and 27 boys) were found to have early breast development; all 170 children were categorized as ≥ Tanner Stage 2 (Table 1). The prevalence of PT among girls and that of GM among boys was 4.8 and 0.8%, respectively (*P* < 0.001). One hundred girls were diagnosed with PT at the age of < 2 years; of these 69 (69.0%) girls experienced spontaneous resolution of PT, while 31 experienced persistence of PT. Twenty-four boys were diagnosed with GM at the age of < 2 years; of these, 10 (41.7%) boys experienced spontaneous resolution of GM. Girls who developed PT before the age of 2 years were more likely to experience spontaneous resolution with passage of time as compared to girls who developed PT at the age of 3–7 years (χ^2^ = 7.75, *P* = 0.005; Fig. 1).

**Table 1:** Age, sex, and area of residence of subjects with early breast development

Group	Frequency (PT and GM)	Percentage (%)
Age (years)		
0–2	124	2.0
2–4	15	0.2
5–7	31	0.5
Sex		
Male (GM)	27 (3295)	0.8
Female (PT)	143 (2978)	4.8
Area of residence		
Urban	102 (3301)	3.1
Suburban	68 (2972)	2.3

*PT* premature thelarche, *GM* gynecomastia

### Relationship between early breast development and physical growth

In the overall study population, the development of PT or GM showed a significant association with height, weight, and waist and/or hip circumference (Table 2). However, no significant association of early breast development with BMI was observed either in girls or boys. The mean BMI of children with and without breast development was 16.10 ± 1.37 kg/m^2^ and 17.70 ± 89.63 kg/m^2^, respectively.

**Table 2:** Relationship between early breast development and physical growth status

Growth status	Subjects without PT/GM	Subjects with PT/GM	F	*t*	*P*
*z Height	−0.18 ± 0.98	0.64 ± 1.41	79.14	−8.538	< 0.001
*z Weight	−0.16 ± 0.98	0.58 ± 1.39	67.88	−7.638	< 0.001
*z Waist perimeter	− 0.01 ± 0.99	0.39 ± 1.30	33.00	−5.156	< 0.001
*z Hip perimeter	−0.21 ± 0.98	0.77 ± 1.44	93.60	−10.204	< 0.001
BMI	17.70 ± 89.63	16.10 ± 1.37	0.12	1.385	0.177

*z: corrected for sex and age

*PT* premature thelarche, *GM* gynecomastia, *BMI* body mass index

### Relationship between early breast development and family environment factors

There was no significant association of early breast development with maternal gestation, birth type, birth weight, feeding history, frequency of exercise by children, and sleep duration (Table 3). Children borne of mothers who had earlier menarche were more likely to develop PT and GM (age at menarche < 12 years, OR = 6.3, 95% CI: 4.4–8.8); the risk of PT and GM was particularly high among children borne of mothers who had menarche by the age of 10 years (OR = 58.2, 95% CI: 33.6–100.7). Family monthly income < 3000 yuan or > 15,000 yuan was associated with an increased risk of PT and GM (OR = 2.2, 95% CI: 0.8–6.2 and OR = 2.4, 95% CI: 1.8–3.3, respectively). Mode of feeding in the first year, exercise frequency, sleep time, or history of major disease was not associated with PT or GM.

**Table 3:** Relationship of early breast development with maternal, gestational and economic factors

Family factors	Subjects without PT/GM *n* (%)	Subjects with PT/GM (%)	OR (95% CI)
Gestation			
Premature birth	412 (97.6)	10 (2.4)	1.0
Term birth	5493 (97.3)	153 (2.7)	1.2 (0.6–2.2)
Post term birth	198 (96.6)	7 (2.7)	1.5 (0.6–3.9)
Birth weight (kg)	3.38 ± 0.71	3.35 ± 0.57	1.0 (0.7–1.3)
Maternal menarche age (years)			
10	29 (47.5)	32 (52.5)	58.2 (33.6–100.7)
< 12	372 (88.4)	49 (11.6)	6.3 (4.4–8.8)
12–13	1462 (97.3)	40 (2.7)	1.4 (1.0–2.1)
> 13	4269 (98.1)	81 (1.9)	1.0
Family monthly income (yuan)			
3000–15,000	4555 (98.0)	94 (2.0)	1.0
< 3000	87 (95.6)	4 (4.4)	2.2 (0.8–6.2)
> 15,000	1461 (95.3)	72 (4.7)	2.4 (1.8–3.3)

*PT* premature thelarche, *GM* gynecomastia, *OR* odds ratio, *CI* confidence interval

### Relationship of early breast development and dietary exposure

Dietary intake of rice, pasta, meat, dairy products, bean products, snacks, drinks, fruits, and vegetables by children was not significantly associated with early breast development (Table 4). Children who consumed greater amount of eggs showed a significantly greater prevalence of PT and GM (F = 41.57, *P* < 0.001).

**Table 4:** Relationship between early breast development and food exposure

Food type	Subjects without PT/GM	Subjects with PT/GM	F	*t*	*P*
Rice (times/week)	6.00 ± 5.81	5.68 ± 5.81	0.31	0.72	0.58
Pasta (times/week)	0.70 ± 1.92	2.73 ± 2.11	0.78	0.18	0.86
Red meat (times/week)	3.77 ± 3.66	3.44 ± 2.91	0.86	1.17	0.24
White meat (times/week)	1.98 ± 2.22	1.71 ± 1.02	0.48	1.63	0.15
Sea food (times/week)	2.36 ± 1.86	2.27 ± 2.15	0.35	0.62	0.55
Eggs (g/week)	22.35 ± 7.68	250.86 ± 31.93	41.57	3.21	< 0.001
Fresh milk (mL/week)	420.73 ± 498.79	408.43 ± 781.75	1.76	0.32	0.19
Milk products (mL/week)	407.78 ± 406.26	484.04 ± 307.31	0.52	0.52	0.47
Fruit juice (mL/week)	298.26 ± 552.05	295.71 ± 202.49	0.19	0.6	0.66
Carbonated drinks (mL/week)	188.01 ± 67.43	183.50 ± 63.29	2.8	0.86	0.09
Honey (mL/week)	194.17 ± 176.92	186.55 ± 77.05	0.26	0.56	0.61
Snacks (times/week)	3.89 ± 3.56	3.89 ± 3.28	0.05	0.09	0.82
Bean products (times/week)	3.16 ± 2.42	2.92 ± 1.97	0.23	1.31	0.63
Vegetables (times/week)	5.17 ± 5.01	4.93 ± 4.45	1.81	0.6	0.18
Fruits (times/week)	4.28 ± 4.14	4.21 ± 3.76	0.45	0.21	0.5

Data presented as mean ± standard deviation

*PT* premature thelarche, *GM* gynecomastia

The resolution of PT and GM was associated with childhood food choices and resolution rates were significantly higher in children who consumed greater amounts of rice (*P* < 0.001), pasta (*P* < 0.001), red meat (*P* < 0.001), fruits (*P* < 0.001), vegetables (*P* < 0.001), and snacks (*P* < 0.001), and lesser amount of eggs (*P* = 0.02) (Table 5).

**Table 5:** Relationship between the resolution of PT/GM and food exposure

Food type	Resolution	No resolution	F	*t*	*P*
Rice (times/week)	7.85 ± 6.39	2.72 ± 3.07	127.7	6.29	< 0.001
Pasta (times/week)	3.17 ± 2.47	2.14 ± 1.30	11.27	3.24	< 0.001
Red meat (times/week)	3.94 ± 3.40	2.68 ± 1.90	20.91	2.84	< 0.001
White meat (times/week)	1.63 ± 1.16	1.64 ± 0.74	3.14	0.66	0.07
Sea food (times/week)	2.52 ± 2.63	1.89 ± 1.14	4.11	1.89	0.44
Eggs (g/week)	23.09 ± 71.34	626.92 ± 4902.61	5.25	1.22	0.02
Fresh milk (mL/week)	424.77 ± 342.94	358.60 ± 159.32	2.37	1.52	0.13
Milk products (mL/week)	559.74 ± 387.16	446.10 ± 328.28	0.67	2.01	0.42
Fruit juice (mL/week)	296.45 ± 223.66	287.12 ± 170.84	0.11	0.3	0.74
Carbonated drinks (mL/week)	173.78 ± 66.30	163.09 ± 57.56	0.38	1.09	0.54
Honey (mL/week)	154.92 ± 77.99	151.08 ± 72.27	0.01	0.33	0.93
Snacks (times/week)	4.56 ± 3.51	2.98 ± 2.69	13.8	3.2	< 0.001
Bean products (times/week)	3.06 ± 2.23	2.60 ± 1.49	6.24	1.54	0.13
Vegetables (times/week)	6.02 ± 5.17	3.35 ± 2.55	24.71	4.04	< 0.001
Fruits (times/week)	5.18 ± 4.33	2.87 ± 2.23	20.26	4.14	< 0.001

Data presented as mean ± standard deviation

*PT* premature thelarche, *GM* gynecomastia

## Discussion

The prevalence of the early breast development in children varies in different populations. In a study by Wang et al., the prevalence of PT and GM in children aged < 8 years was 2.4% in urban areas and 1.0% in the suburbs of Shanghai, China [15]. Similarly, the prevalence of PT in girls aged 6–8 years was 16.0% in Haikou City [16]. In the present study, the prevalence of PT and GM in children aged 2–7 years in Zhejiang province was 4.8 and 0.8%, respectively. Our findings are consistent with those reported from Shanghai but different from those reported from Haikou. The prevalence of PT in the present study is lower than that reported from Turkey (8.9%) [2] and Italy (11%) [3], but approximates the rates reported from Denmark (3%) [4] and the US (4.7%) [5, 6].

Early breast development in young children is a benign and self-limiting phenomenon that typically regresses spontaneously with age. The progression of PT to central precocious puberty (CPP) is quite uncommon. Studies have provided very different estimates as to how often progression of PT to CPP occurs with a recent study [17] reporting only 2% of girls progress while 2 other studies [18, 19] reported that 18–20% progressing from PT to CPP. Persistence of PT or progression of PT to CPP signifies a more complicated condition in early childhood. The reported rates of spontaneous regression of early breast development range from 50.5–69.5% [20]. In a study of 91 Taiwanese girls with PT, 57.6% girls experienced spontaneous resolution of PT, while 19% experienced progression to CPP [21]. In the present study, the resolution rate of PT in girls was 61.5% (88 of 143) and that of GM in boys was 37.0% (10/27).

We also found that the resolution rate of PT among girls aged 0–2 years was significantly higher than that among girls aged > 3 years (*P* = 0.005); this phenomenon may be attributable to the so-called mini-puberty theory. After delivery, the hypothalamo-pituitary-gonadal axis is activated by the low levels of estrogen in newborns, which leads to pubertal levels of estrogen (also referred to as mini-puberty) [22]. In girls, the mini-puberty lasts for about 2–3 years followed by its spontaneous resolution; this explains the higher rates of incidence and resolution of PT in this age-group. The mini-puberty lasts for about 6 months after birth in boys, during which time the long-term testicular functions and sperm production are regulated; this contributes to masculinization of the brain. We postulate that if the mini-puberty is somehow interfered in boys, the gynecomastia would probably occur and persist as found in the current study.

Some studies have reported an association between PT and BMI. In a study by Zeynep et al., the occurrence of PT among Turkish girls with normal BMI was 3.2% as against 12.3% among girls with BMI above the 85th percentile [2]. However, others have argued that higher BMI may be a consequence rather than a determinant of PT [23, 24] . In the current study, the physical growth indices including BMI were not found to be associated with PT or GM; this discrepancy may be attributable to ethnicity-related factors. Zeynep et al. also reported that among girls with normal BMI, only 1.3% of non-Hispanic white girls had PT whereas the prevalence of PT in non-Hispanic black and Mexican American girls was 12.1 and 19.2%, respectively.

Maternal age at menarche may provide some insights into the role of genetic factors on PT and puberty. Some studies have found an association between maternal age at menarche and PT [17, 25], while others have found no such association [2, 26]. In the current study, we found a significant association of maternal age at menarche with PT and GM. Children whose mothers experienced menarche at the age of < 12 years were more likely to develop PT or GM in early childhood.

In the present study, the intake of eggs was associated with both PT and GM. Nutritional factors have been frequently considered as putative agents that could influence early breast development in children. Bratberg et al. reported that children with surplus dietary intake were more likely to develop PT or GM [27]. Gunther et al. found that children aged 5–6 years who regularly consumed animal proteins were at a higher risk of early breast development [28]. However, other studies found no significant association of intake of milk and eggs with early breast development [2] or between consumption of soya bean derived products and PT [20, 29].

Environmental endocrine disrupting chemicals and oestrogen-like agents have often been suggested as underlying causes of early breast development in young children [30–32]. These agents most likely influence the children through contamination of food. There is widespread concern about the illegal usage of endocrine disrupting chemicals in poultry, dairy, and fish farms. Given the poor regulation of Chinese poultry farms, these harmful agents may pollute the eggs and exposure of children to such eggs may increase the prevalence of PT and GM.

In the current study, we found a significant association of PT and GM with family income. Children belonging to families whose monthly income was either < 3000 yuan or > 15,000 yuan had a higher risk of PT and GM as compared to their counterparts with a family income of 3000–15,000 yuan. These results may suggest that early breast development in children from poor families may be linked to greater exposure to environmental pollutants while in children from rich families, the association may be attributable to surplus nutrition and/or greater intake of endocrine disrupting chemicals and oestrogen-like agents through animal proteins.

Some limitations of our study need to be considered. First, although the study was conducted by trained endocrinologists, the inter-observer variability with respect to examination findings cannot be ruled out. Second, distinction between girls with PT and those with Gonadotropin-releasing hormone (GnRH)-dependent precocious puberty may be challenging especially among younger girls [33, 34]; long term follow-up is required to distinguish between these concitions.

In summary, we found that dietary factors may influence early breast development in young children. Environmental contamination of food with endocrine disrupting chemicals and oestrogen-like agents may affect the children. Premature thelarche among girls aged < 2 years was more likely to be resolved than those developed PT at a later age. While once the gynecomastia occurred in boys, it was more likely to remain. The discrepancy in the reported correlates of early breast development in children suggests the need for further investigations to provide more definitive evidence.

## Conclusions

This study examined the prevalence of premature thelarche among girls and that of gynecomastia among boys in Southern China. The prevalence rates approximated those reported from some other countries. The socioeconomic status of family, early onset of menarche in mother, and consumption of eggs were strongly associated with premature breast development in early childhood. Greater attention should be paid to the diet of children and adolescents to prevent breast development.

## Abbreviations

CIs: Confidence intervals
GM: Gynecomastia
ORs: Odds ratios
PT: Premature thelarche
